# Socioeconomic Inequalities in Oral Frailty Among Older Adults: A Causal Mediation Analysis on the Role of Prevention From Tooth Loss

**DOI:** 10.1111/joor.70042

**Published:** 2025-08-27

**Authors:** Mieko Fujita, Kenji Takeuchi, Yudai Tamada, Taro Kusama, Tatsuo Yamamoto, Katsunori Kondo, Ken Osaka

**Affiliations:** ^1^ Department of International and Community Oral Health Tohoku University Graduate School of Dentistry Sendai Miyagi Japan; ^2^ Department of Dental Hygiene, Junior College University of Shizuoka Shizuoka Shizuoka Japan; ^3^ Department of Preventive Medicine Nagoya University Graduate School of Medicine Nagoya Aichi Japan; ^4^ Division of Statistics and Data Science, Liaison Center for Innovative Dentistry Tohoku University Graduate School of Dentistry Sendai Miyagi Japan; ^5^ Department of Preventive Dentistry and Dental Public Health Kanagawa Dental University Yokosuka Kanagawa Japan; ^6^ Department of Social Preventive Medical Sciences, Center for Preventive Medical Sciences Chiba University Kashiwa Chiba Japan; ^7^ Department of Gerontological Evaluation, Center for Gerontology and Social Science, Research Institute National Center for Geriatrics and Gerontology Obu Aichi Japan

**Keywords:** frailty, Japan, mediation analyses, social class, tooth loss

## Abstract

**Background:**

Although social inequalities in oral health have been suggested, the link between socioeconomic status (SES) and oral frailty (OF) remains unclear. We aimed to investigate the inequalities in OF according to SES and the extent to which inequalities are mitigated by preventing tooth loss.

**Methods:**

We used cross‐sectional data from 21 542 functionally independent participants aged ≥ 65 (48.5% men) from the Japan Gerontological Evaluation Study. The prevalence of OF, number of teeth, and educational attainment (EA) and equivalent income (EI) were used as outcome, mediator and explanatory variables, respectively. A Poisson regression model was used to examine the association between SES and OF. Causal mediation analysis was performed to calculate the prevalence ratios (PRs) and 95% confidence intervals (CIs) of the controlled direct effects (CDEs) of the number of teeth. The proportion eliminated (PE) of the ≥ 20 remaining teeth was calculated.

**Results:**

Overall 7984 participants had OF. The prevalence of OF was 1.45 times higher in participants with ≤ 9 years of EA and 1.38 times higher in participants with an EI of < $20.000. The estimated total effect (TE) of low EA or low EI on the prevalence of OF was mediated by the number of teeth (TE PR, 1.30 [95% CI, 1.25–1.35]; CDE PR, 1.22 [95% CI, 1.10–1.33]; PE, 28.2%) or (TE PR, 1.26 [95% CI, 1.22–1.31]; CDE PR, 1.23 [95% CI, 1.14–1.32]; PE, 12.9%).

**Conclusion:**

OF showed a clear social gradient based on SES. However, this association could be mediated by the remaining ≥ 20 teeth.

## Graphical abstract

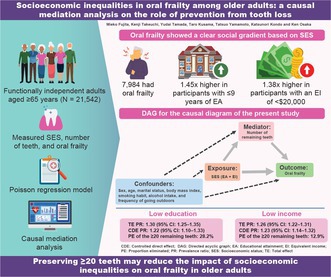

## 1 Introduction

Oral frailty is a relatively recent concept in geriatric oral health, referring to the early stage of declining oral function characterised by mild symptoms such as choking or chewing difficulty that may increase the risk of adverse health outcomes, including physical frailty and disability [1, 2]. Recent Japanese studies on community‐dwelling older adults indicated that those with oral frailty had a significantly higher risk of physical frailty, sarcopenia, functional disability, new‐onset mild cognitive impairment, long‐term care and mortality [3, 4] than those without oral frailty. A recent study from Taiwan reported that oral frailty was associated with an increased risk of physical frailty [5]. By contrast, oral frailty is considered a modifiable risk factor [3]; simply put, oral frailty is a reversible condition. A previous study conducted among community‐dwelling older adults reported that the implementation of a 12‐week oral frailty programme in an intervention group significantly improved the components of oral frailty, specifically oral motor skills for/ta/and tongue pressure [6]. Thus, oral frailty is both a risk factor and a modifiable factor for several adverse health outcomes in older people. Thus, early detection and intervention in individuals at high risk of oral frailty are crucial.

Some systematic reviews have suggested the existence of social inequalities in oral health [7, 8, 9, 10]. A clear relationship was found between socioeconomic status (SES) and oral health outcomes, indicating that individuals with a lower SES are at an increased risk of dental caries [7, 10], periodontitis [8] and tooth loss [9]. Among older adults, lower income [11] and educational attainment [12] were significantly associated with a higher risk of total tooth loss. However, to the best of our knowledge, no study has reported the social gradients in oral frailty. Furthermore, regarding the components of oral frailty, only one study assessed the difference in subjective chewing difficulty according to income and educational attainment status [13].

The socioeconomic inequalities in the prevalence of oral frailty may be partially explained by the socioeconomic gradient in oral health status deterioration, particularly a low number of remaining teeth. A significant social gradient exists in the number of remaining teeth in older age groups [14]. The number of remaining teeth is also strongly associated with masticatory ability, a component of oral frailty [15]. In addition, a small number of remaining teeth is associated with poor oral function due to the diminished masticatory ability [16] and limited food choices [17]. Thus, the number of remaining teeth may mediate the association between SES and oral frailty. Reducing health inequalities is a global agenda [18], and it is necessary to identify inequalities in oral frailty risk due to SES in order to consider oral frailty strategies. Recently, causal mediation analyses have been conducted to explore the modifiable mediators (e.g., number of remaining teeth) instead of the exposure factors, which are difficult to intervene (e.g., SES) [19].

Using data from a large epidemiological study, this cross‐sectional study aimed to determine the presence of inequalities in the prevalence of oral frailty according to SES. Additionally, this study aimed to determine the extent to which these inequalities could be mitigated by preventing tooth loss. Consequently, we aimed to test the following two hypotheses: (1) whether a socioeconomic gradient exists in the prevalence of oral frailty and (2) whether preventing tooth loss would help reduce the inequalities in oral frailty in old age.

## 2 Methods

### 2.1 Study Design and Participants

This study was based on data from the Japan Gerontological Evaluation Study (JAGES), an ongoing cohort study exploring the social determinants of health among individuals aged ≥ 65 years [20, 21]. We used cross‐sectional data from the 2019 wave, conducted between December 2019 and January 2020. During this period, self‐reported questionnaires were sent to 345 356 independent people aged ≥ 65 years, who did not receive long‐term care insurance benefits and were randomly recruited or completely enumerated from 60 municipalities in Japan. A random sample of official residents registered in 43 large municipalities and a complete census of older residents in the remaining 17 smaller municipalities were obtained. A total of 240 889 individuals responded to our survey, yielding a response rate of 69.8% (range: 54.4%–89.8% from the 60 municipalities). Eight versions of the JAGES questionnaire were used during the survey. Each version comprised a different subset of questions with a common set of core questions. One‐eighth of the participants were randomly assigned to answer each subset of questions. A total of 31 747 participants responded to questions regarding oral health, which were distributed to one‐eighth of the randomly allocated participants. After excluding respondents who did not demonstrate independence in performing the activities of daily living, did not provide informed consent, or had no available data on sex or age, 15 372 (men, 8065; women, 7307) functionally independent participants were included in the analyses.

### 2.2 Outcome Assessment

The outcome of this study was oral frailty, assessed based on a slightly modified version of the Oral Frailty Index‐8 (OFI‐8), a validated screening tool proposed by Tanaka et al. for identifying older adults at risk of oral frailty [22]. The self‐reported questionnaires used for assessment comprised seven questions, which were rated as follows: ‘Do you have any difficulties eating tough foods compared with six months ago? (Yes: 2 points)’, ‘Have you recently experienced choking while consuming tea or soup? (Yes: 2 points)’, ‘Do you use dentures? (Yes: 2 points)’, ‘Do you frequently experience dry mouth? (Yes: 1 point)’, ‘Do you go out less frequently than you did last year? (Yes: 1 point)’, ‘How many times do you brush your teeth in a day? (< 2 times/day: 1 point)’ and ‘Do you visit a dental clinic at least annually?’ (No: 1 point). The total score ranged from 0 to 10 points. The primary outcome measure for the analysis was oral frailty, which was defined as a total score of 4 or more points.

### 2.3 Exposure Assessment

Two SES indicators were assessed as exposure variables: educational attainment and equivalised household income. Educational attainment was divided into three categories: ≤ 9 years (junior high school or lower secondary education), 10–12 years (high school or upper secondary education) and ≥ 13 years (college or university). Equivalised household income per year was calculated by dividing the annual household income by the square root of the number of people living together. It was divided into three categories: < 2.00, 2.00–3.99, or ≥ 4.00; 10 000$, 1 US$ = 100 JPY. Based on the methodology used in previous mediation analysis studies [23, 24], we created binary variables representing low SES (≤ 9 years/≥ 10 years; < 2 million $/≥ 2 million $) for causal mediation analyses.

### 2.4 Mediator Assessment

The number of remaining teeth at baseline was used as the mediator variable. The validity of self‐reported tooth count has been reported in a previous study [25]. The participants were asked the following question: ‘What is the status of your dental health?’ Then, they were requested to choose a response from the following options: ‘I have 20 or more natural teeth’, ‘I have 10–19 natural teeth’, ‘I have 5–9 natural teeth’, ‘I have 1–4 natural teeth’, or ‘I have no natural teeth’. These answers were further dichotomized into two categories: ‘having 20 or more remaining teeth’ and ‘having ≤ 19 remaining teeth’. The cut‐off at < 20 remaining teeth is widely used in epidemiological studies. This cut‐off point was determined based on the relationships of the number of teeth and oral function with longevity [26], and the findings of previous studies that investigated the association between oral and systemic health using self‐reported questionnaires [27, 28, 29]. For the sensitivity analysis, we also conducted the causal mediation analysis using severe tooth loss (‘having 10 or more remaining teeth’ vs. ‘having ≤ 9 remaining teeth’) as the mediator.

### 2.5 Other Covariates

Based on previous studies and clinical knowledge [13, 14, 30, 31], we controlled for the following potential confounders: sex (men or women), age (65–69, 70–74, 75–79, 80–84, or ≥ 85 years), marital status (married or not married), body mass index (< 18.5, 18.5–24.9, 25.0–29.9, or ≥ 30.0), smoking habit (current, former, or never), alcohol intake (current, former, or never) and frequency of going outdoors (4 or more times a week, 2–3 times a week, or less than once a week).

### 2.6 Statistical Analysis

To calculate the adjusted prevalence ratios (PRs) and their corresponding 95% confidence intervals (95% CIs) of the prevalence of oral frailty according to the identified SES indicators, a modified Poisson regression model (Poisson regression with sandwich standard errors) was used.

**FIGURE 1:**
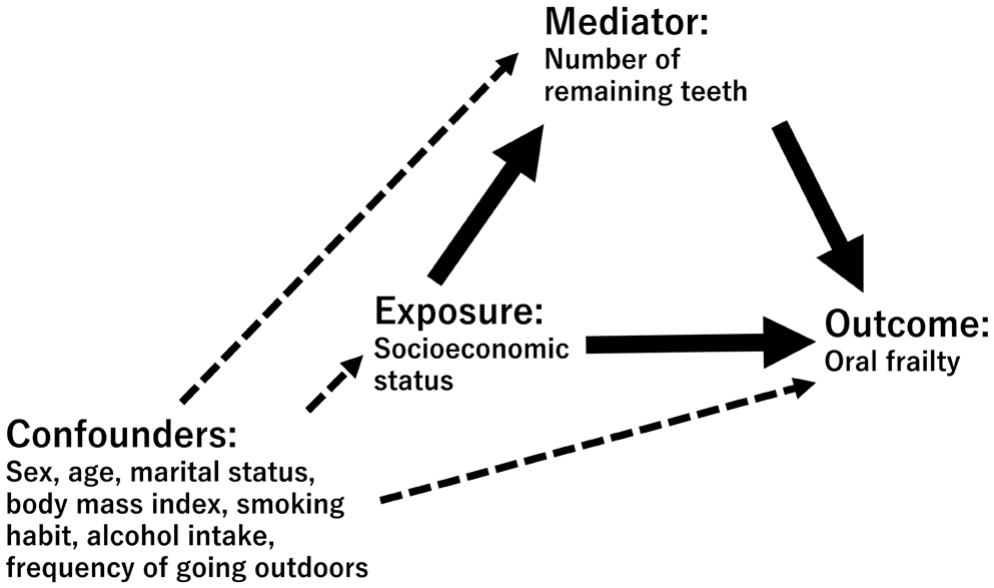
Directed acyclic graph for the causal diagram of the present study. The effect of socioeconomic status on oral frailty risk is mediated by the number of remaining teeth. Potential confounders of the relationship between exposure, outcome and mediators are considered.

Causal mediation analyses were used to calculate the total effect (TE), controlled direct effect (CDE) and proportion eliminated (PE) to evaluate the protective effect of maintaining at least 20 remaining teeth against the risk of oral frailty [19, 32]. A causal diagram of the analytical model is shown in Figure 1. CDE (*m*) captured the effect of exposure on the outcome if the mediator was set at level ‘*m*’. We calculated the CDE (*m* = 1) (having 20 or more remaining teeth), which refers to a condition in which all participants had 20 or more remaining teeth. The PEs (*m* = 1) are used for performing policy measures and indicate the amount of the effect of exposure on the outcome that could be eliminated by intervening at level ‘*m*’. PEs (*m* = 1) were calculated using the following formula: PE (*m* = 1) = ([PR^TE^—PR^CDE (*m* = 1)^]/[PR^TE^‐1]). PE indicates how much of the effect of exposure on outcomes could be eliminated by intervening on intermediate factors and is important from a policy perspective [33]. In this study, PE was calculated because the objective was to examine factors that mitigate the oral frailty risk inequality due to SES. The Stata command ‘*paramed*’ was used for performing a causal mediation analysis [33]. The mediator, the number of remaining teeth (‘having 20 or more remaining teeth/having 19 or less remaining teeth’) was used as a binary variable.

We conducted multiple imputations using chained equations to impute the missing data on all variables and generated 20 imputed datasets according to the recommendations of a previous study [34]. The estimates for each imputed dataset were combined using Rubin's rule. As part of the sensitivity analysis, complete case analyses were conducted to compare the estimates with those obtained from the imputed datasets. Although confounders were included as far as possible in the analysis, it is possible that unmeasured confounders may influence the present results. Therefore, to identify the strength of the unmeasured covariates that affected the estimates, we calculated the mediational E‐values of CDE. The mediational E‐values of CDE indicate the minimum strength of the association that an unmeasured confounder needs to be present in both the mediator and outcome under the conditions of the measured covariates to explain the mediating effect [35]. All statistical analyses were performed using Stata (version 17.0; Stata Corp, College Station, TX, USA), and a two‐sided *p*‐value < 0.05 was considered significant in all cases. This study was reported based on the Strengthening the Reporting of Observational Studies in Epidemiology (STROBE) guidelines.

### 2.7 Ethical Considerations

The JAGES protocol and informed consent procedure were approved by the Ethics Committees of the National Centre for Geriatrics and Gerontology (approval number: 992–3) and Chiba University (approval number: 2493). The participants were informed that participation in the study was voluntary and that completing and returning the questionnaire was interpreted as informed consent.

## 3 Results

Table 1 shows the characteristics of the study population according to the oral frailty status. After multiple imputations, 21 542 participants (48.5% men) were analysed. The prevalence of oral frailty was 37.1%. Approximately 24.4%, 44.2% and 31.4% of the participants had ≤ 9, 10–12 and ≥ 13 years of educational attainment, respectively. Moreover, 47.8%, 39.8% and 12.4% of the participants had equivalent incomes of < $20 000, $20 000–40 000 and > $40 000, respectively. Among all participants, 43.6% had ≤ 19 remaining teeth. Approximately 48.5%, 36.4% and 29.1% of the participants with ≤ 9, 10–12 and ≥ 13 years of educational attainment, respectively, had oral frailty. Furthermore, oral frailty was prevalent in 42.9%, 32.8% and 28.2% of patients with equivalent incomes of < $20 000, $20 000–40 000 and > $40 000, respectively. The characteristics of the study population before multiple imputations are shown in Table S1.

**TABLE 1:** Characteristics of the study population according to oral frailty status (*n* = 21 542).

	Total (*n* = 21 542)		Oral frailty status			
			No (*n* = 13 558)		Yes (*n* = 7984)	
	*n*	%	*n*	%	*n*	%
Sex						
Men	10 458	48.5	6313	60.4	4145	39.6
Age, years						
65–69	5401	25.1	3904	72.3	1497	27.7
70–74	6413	29.8	4274	66.6	2139	33.4
75–79	5298	24.6	3216	60.7	2082	39.3
80–84	3039	14.1	1587	52.2	1452	47.8
≤ 85	1391	6.5	577	41.5	814	58.5
Marital status						
Married	15 877	73.7	10 283	64.8	5593	35.2
Body mass index						
< 18.5	1467	6.8	858	58.5	609	41.5
18.5–24.9	14 666	68.1	9384	64.0	5283	36.0
25.0–29.9	4861	22.6	3029	62.3	1832	37.7
≥ 30.0	548	2.5	287	52.5	260	47.5
Smoking habit						
Current	2204	10.2	1135	51.5	1069	48.5
Former	6683	31.0	3991	59.7	2692	40.3
Never	12 655	58.7	8433	66.6	4222	33.4
Alcohol intake						
Current	9032	41.9	5805	64.3	3227	35.7
Former	2253	10.5	1237	54.9	1016	45.1
Never	10 257	47.6	6516	63.5	3741	36.5
Frequency of going outdoors						
4 or more times a week	16 477	76.5	10 999	66.8	5477	33.2
2–3 times a week	3674	17.1	1962	53.4	1712	46.6
Less than once a week	1391	6.5	597	42.9	794	57.1
Equivalent income (1$ = 100JPY)						
< 20 000	10 294	47.8	5876	57.1	4419	42.9
20 000–40 000	8584	39.8	5769	67.2	2815	32.8
> 40 000	2664	12.4	1914	71.8	750	28.2
Educational attainment, years						
≤ 9	5262	24.4	2710	51.5	2552	48.5
10–12	9517	44.2	6056	63.6	3461	36.4
≥ 13	6763	31.4	4792	70.9	1971	29.1
Number of remaining teeth						
≤ 19	9395	43.6	3850	41.0	5546	59.0
≥ 20	12 147	56.4	9709	79.9	2438	20.1

*Note:* Each value is the mean of 20 imputed datasets.

Table 2 presents the results of the Poisson regression analysis of the association between oral frailty and SES. Lower SES was significantly associated with a higher PR for oral frailty in both crude and fully adjusted models. Even after adjusting for confounders, the prevalence of oral frailty was 1.45 times (95% CI, 1.38–1.53) higher in participants with ≤ 9 years of educational attainment than in those with ≥ 13 years. In addition, the prevalence was 1.38 (95% CI, 1.28–1.49) times higher in participants with an equivalent income of < $20 000 than in those with > $40 000. Similar results were obtained in the complete case analysis (Table S2).

**TABLE 2:** Association between the socioeconomic indicators and the prevalence of oral frailty (*n* = 21 542).

	Crude PR (95% CI)	Adjusted PR (95% CI)^a^
Equivalised income, $		
< 20 000	1.52 (1.41–1.64)	1.38 (1.28–1.49)
20 000–40 000	1.16 (1.08–1.26)	1.14 (1.05–1.23)
> 40 000	1.00 (reference)	1.00 (reference)
Educational attainment, years		
≤ 9	1.66 (1.58–1.75)	1.45 (1.38–1.53)
10–12	1.25 (1.19–1.31)	1.22 (1.17–1.28)
≥ 13	1.00 (reference)	1.00 (reference)

Abbreviations: CI, confidence interval; PR, prevalence ratio.

^a^ Adjusted for sex, age, marital status, body mass index, smoking habit, alcohol intake and frequency of going outdoors.

Table 3 shows the results of the causal mediation analysis, showing the TE and CDE of low educational attainment (≤ 9 years) and low equivalised income (< $20 000) on the prevalence of oral frailty when the mediator (*m*) was fixed at *m* = 1 (number of remaining teeth: > 20). The estimated TE of low educational attainment or low equivalised income was mediated by the number of remaining teeth [TE PR, 1.30 (1.25–1.35); CDE PR, 1.22 (1.10–1.33); PE, 28.2%] or [TE PR, 1.26 (1.22–1.31); CDE PR, 1.23 (1.14–1.32); PE, 12.9%]. The sensitivity analysis with complete cases also suggested similar results (Table S3). Additionally, Table S4 shows the results of the sensitivity analysis using severe tooth loss as the mediator. Compared to the results when the cut‐off point was set at < 20 remaining teeth, the PE decreased, but a similar trend was observed. The calculated mediational E‐values suggested that the unmeasured confounder needed to associate with both mediator and outcome to explain the CDE of the number of remaining teeth by 1.7‐ to 1.8‐fold in the prevalence ratio scale conditional on included covariates (Table S5).

**TABLE 3:** The contribution of the number of remaining teeth in explaining the association between socioeconomic status and oral frailty.

Explanatory variables^a^	Mediator	TE, PR (95% CI^b^)	CDE, PR (95% CI^b^)	PE^c^ (%)
Equivalised income, $ (ref, ≥ 20 000)	Number of remaining teeth: ≥ 20	1.26 (1.22–1.31)	1.23 (1.14–1.32)	12.9
Educational attainment, years (ref, ≥ 10)		1.30 (1.25–1.35)	1.22 (1.10–1.33)	28.2

*Note:* All models were adjusted for sex, age, marital status, body mass index, smoking habits, alcohol intake and frequency of going out.

Abbreviations: 95% CI, 95% confidence interval; CDE, controlled direct effect; PE, proportion eliminated; PR, prevalence ratio; TE, total effect.

^a^ Each explanatory variable was separately included.

^b^ Estimated by bootstrap with 1000 replications.

^c^ Proportion eliminated = (PR^TE^—PR^CDE^)/(PR^TE^—1).

## 4 Discussion

In this study of community‐dwelling older adults, we found a clear social gradient in oral frailty based on SES. In other words, individuals with lower equivalent income and educational attainment were at an increased risk of oral frailty. However, this association was mitigated by the presence of 20 or more teeth. Our findings suggest that the prevention of tooth loss may reduce the risk of oral frailty due to lower SES.

Importantly, although several studies have reported the existence of social gradients in physical frailty based on SES [36], no previous studies have investigated the association between SES and oral frailty. Therefore, this study is the first to report the social gradients of oral frailty based on SES. In relation to the components of oral frailty, two previous studies investigated the association between SES and chewing ability. According to a study in South Korea, respondents aged ≥ 60 years with lower income or educational attainment tended to have a higher risk of a decline in chewing ability. In addition, the social gradient of educational attainment is stronger than that of income [13]. Similarly, another study conducted in Brazil with 890 individuals aged ≥ 60 years showed that lower individual income was associated with a higher risk of a decline in chewing ability [37]. These findings are in agreement with those of our study, although we focused on oral frailty as a decline in the overall oral function and not just a decline in chewing ability.

Our study indicated that having fewer than 20 teeth is an important mediating factor for SES and oral frailty, and at least 10% of this association was explained. In other words, a decrease in the number of remaining teeth could be a background factor in the transition from healthy oral function to oral frailty. Preventing tooth loss is important to avoid the transition to oral frailty. This implies that, from a clinical and public health perspective, promoting preventive dental care—such as regular scaling and fluoride application—can reduce SES‐related disparities in oral frailty by targeting the underlying causes of tooth loss. The current results are in line with those of a previous systematic review, which showed that tooth loss has a significant negative impact on chewing ability, a component of oral frailty [15]. Our findings are also in line with those of previous studies, which reported that participants with lower SES were at an increased risk of tooth loss [9, 11, 12]. In addition, SES is positively associated with the use of preventive dental care [38] and negatively associated with poor oral health behaviours [39]. Furthermore, the lack of preventive dental care and a high concentration of poor oral health behaviours due to low SES might cause dental caries and periodontal disease, and in turn lead to a reduced number of remaining teeth in older age. As preventive dental care is not covered by the Japanese insurance system, the relative cost burden of preventive dental care is higher among those with lower SES, which may be a barrier to dental visits. Therefore, to reduce inequality in oral frailty due to differences in SES, a public health approach that is not dependent on SES, such as water fluoridation and taxes on sugar, should be implemented to prevent tooth loss.

In our study, the association between educational attainment and oral frailty was more strongly mediated by the number of remaining teeth than the association between equivalent income and oral frailty. The number of remaining teeth in old age is a cumulative result of an individual's life‐course [40]. Income is a short‐term changeable indicator [41] and mainly reflects current SES; however, educational attainment captures the long‐term effects of early life circumstances on health [42] and mainly reflects the SES of an individual's life course. Therefore, the number of remaining teeth in older age, as determined by life course accumulation, is more likely to be influenced by educational attainment than by income. Additionally, part of the equivalent income of older adults may have been supplemented by pensions, which may have led to a smaller effect on oral frailty risk, compared with educational attainment. Furthermore, a social gradient in the number of remaining teeth based on SES was greater for educational attainment than for income [13].

This study has some limitations. First, this was a cross‐sectional study, and the temporality between SES, number of teeth and oral frailty cannot be established. Causal mediation analysis is ideally performed with longitudinal data to strengthen causal inferences. While the directionality assumed in our analysis is supported by prior studies [7, 8, 9, 10], reverse causation cannot be entirely ruled out. Future longitudinal studies are needed to validate our findings. Despite these limitations, the application of causal mediation analysis in cross‐sectional designs has precedents in the literature [43, 44] and may still offer useful preliminary insights into potential intervention points. Second, owing to the use of a modified version of the OFI‐8, one of the eight original OFI‐8 questions could not be assessed. Although the original version of the questionnaire defined oral frailty as a score of 4 or more on an 11‐point scale, we used a score of 4 or more on a 10‐point scale to indicate oral frailty. This may have led to an underestimation of the number of participants with oral frailty. However, this situation did not affect the association between SES and oral frailty observed in the present study. Third, although we adjusted for the widest possible range of confounders, unmeasured, plausible confounders may be residual confounders. The calculated mediating E values suggest that CDE for number of teeth is not explained if there is unmeasured confounding and if that confounding factor is associated with both number of teeth and oral frailty by a magnitude greater than 1.7 on the prevalence ratio scale, conditional on the included covariates.

In conclusion, this study found that the prevalence of oral frailty increases with lower SES in an older population in Japan. However, the inequality in oral frailty based on SES could be partially mediated by having 20 or more remaining teeth.

## Author Contributions

M.F., T.K. and K.T. were involved in study design and data interpretation. M.F., K.T., T.K. and K.K. collected and verified the data. M.F., T.K. and K.T. were involved in data analysis. M.F. and K.T. wrote the initial draft of this manuscript. K.T., Y.T., T.K., T.Y., K.K. and K.O. supervised and administered the study. K.T., T.Y., K.K. and K.O. acquired the funding. All authors provided their final approval and agreed to be accountable for all aspects of the study.

## Conflicts of Interest

The authors declare no conflicts of interest.

## Supporting information


**Data S1:** joor70042‐sup‐0001‐DataS1.docx.

## Data Availability

The data that support the findings of this study are available on request from the corresponding author. The data are not publicly available due to privacy or ethical restrictions.
