# Supplementary material for: Socioeconomic Inequalities in Oral Frailty Among Older Adults: A Causal Mediation Analysis on the Role of Prevention From Tooth Loss
Source: J Oral Rehabil. 2025 Aug 27;52(12):2412–9. doi: 10.1111/joor.70042 (PMC12624153; doi:10.1111/joor.70042)
Supplement: Supplementary file 1 — Data S1: joor70042‐sup‐0001‐DataS1.docx. [file JOOR-52-2412-s001.docx]

**Supplementary materials**

**Tables:**

Table S1. Characteristics of the study population according to oral frailty status assessed using complete case analysis (n = 15,372). 2

Table S2. Association between the socioeconomic indicators and the prevalence of oral frailty assessed using complete case analysis (n = 15,372). 4

Table S3. The contribution of number of remaining teeth in explaining the association between socioeconomic status and oral frailty assessed using complete case analysis (n = 15,372) 5

Table S4. The contribution of number of remaining teeth in explaining the association between socioeconomic status and oral frailty assessed based on a different cutoff for the number of remaining teeth 6

Table S5. Robustness to unmeasured confounding (Mediational E-values) of Mediating effect due to number of remaining teeth in the association between socioeconomic status and oral frailty 7

# Table S1. Characteristics of the study population according to oral frailty status assessed using complete case analysis (n = 15,372).

|  | **Total (n = 15,372)** | |  | **Oral frailty status** | | | | |
| --- | --- | --- | --- | --- | --- | --- | --- | --- |
|  |  |  |  | **No (n = 10,003)** | |  | **Yes (n = 5,369)** | |
|  | **n** | **%** |  | **n** | **%** |  | **n** | **%** |
| Sex |  |  |  |  |  |  |  |  |
| Men | 8,065 | 52.5 |  | 5,023 | 62.3 |  | 3,042 | 37.7 |
| Age, years |  |  |  |  |  |  |  |  |
| 65–69 | 4,190 | 27.3 |  | 3,062 | 73.1 |  | 1,128 | 26.9 |
| 70–74 | 4,821 | 31.4 |  | 3,285 | 68.1 |  | 1,536 | 31.9 |
| 75–79 | 3,670 | 23.9 |  | 2,267 | 61.8 |  | 1,403 | 38.2 |
| 80–84 | 1,930 | 12.6 |  | 1,062 | 55.0 |  | 868 | 45.0 |
| ≤85 | 761 | 5.0 |  | 327 | 43.0 |  | 434 | 57.0 |
| Marital status |  |  |  |  |  |  |  |  |
| Married | 11,698 | 76.1 |  | 7,773 | 66.4 |  | 3,925 | 33.6 |
| Body mass index |  |  |  |  |  |  |  |  |
| <18.5 | 978 | 6.4 |  | 599 | 61.2 |  | 379 | 38.8 |
| 18.5–24.9 | 10,474 | 68.1 |  | 6,929 | 66.2 |  | 3,545 | 33.8 |
| 25.0–29.9 | 3,534 | 23.0 |  | 2,266 | 64.1 |  | 1,268 | 35.9 |
| ≥30.0 | 386 | 2.5 |  | 209 | 54.1 |  | 177 | 45.9 |
| Smoking habit |  |  |  |  |  |  |  |  |
| Current | 1,649 | 10.7 |  | 873 | 52.9 |  | 776 | 47.1 |
| Former | 5,123 | 33.3 |  | 3,161 | 61.7 |  | 1,962 | 38.3 |
| Never | 8,600 | 55.9 |  | 5,969 | 69.4 |  | 2,631 | 30.6 |
| Alcohol intake |  |  |  |  |  |  |  |  |
| Current | 6,876 | 44.7 |  | 4,545 | 66.1 |  | 2,331 | 33.9 |
| Former | 1,585 | 10.3 |  | 902 | 56.9 |  | 683 | 43.1 |
| Never | 6,911 | 45.0 |  | 4,556 | 65.9 |  | 2,355 | 34.1 |
| Frequency of going outdoors |  |  |  |  |  |  |  |  |
| 4 or more times a week | 12,061 | 78.5 |  | 8,260 | 68.5 |  | 3,801 | 31.5 |
| 2–3 times a week | 2,463 | 16.0 |  | 1,364 | 55.4 |  | 1,099 | 44.6 |
| Less than once a week | 848 | 5.5 |  | 379 | 44.7 |  | 469 | 55.3 |
| Equivalent income  (1$ = 100JPY) |  |  |  |  |  |  |  |  |
| <20,000 | 6,943 | 45.2 |  | 4,118 | 59.3 |  | 2,825 | 40.7 |
| 20,000–40,000 | 6,415 | 41.7 |  | 4,417 | 68.9 |  | 1,998 | 31.1 |
| >40,000 | 2,014 | 13.1 |  | 1,468 | 72.9 |  | 546 | 27.1 |
| Educational attainment, years |  |  |  |  |  |  |  |  |
| ≤9 | 3,193 | 20.8 |  | 1,732 | 54.2 |  | 1,461 | 45.8 |
| 10–12 | 6,926 | 45.1 |  | 4,490 | 64.8 |  | 2,436 | 35.2 |
| >13 | 5,253 | 34.2 |  | 3,781 | 72.0 |  | 1,472 | 28.0 |
| Number of remaining teeth |  |  |  |  |  |  |  |  |
| <19 | 6,302 | 41.0 |  | 2,687 | 42.6 |  | 3,615 | 57.4 |
| >20 | 9,070 | 59.0 |  | 7,316 | 80.7 |  | 1,754 | 19.3 |

# Table S2. Association between the socioeconomic indicators and the prevalence of oral frailty assessed using complete case analysis (n = 15,372).

|  | **Crude PR (95% CI)** | **Adjusted PR (95% CI)^*^** |
| --- | --- | --- |
| Equivalized income, $ |  |  |
| <20,000 | 1.50 (1.39–1.62) | 1.37 (1.27–1.48) |
| 20,000–40,000 | 1.15 (1.06–1.24) | 1.13 (1.04–1.22) |
| >40,000 | 1.00 (reference) | 1.00 (reference) |
| Educational attainment, years |  |  |
| ≤9 | 1.63 (1.54–1.73) | 1.44 (1.35–1.52) |
| 10–12 | 1.26 (1.19–1.32) | 1.24 (1.17–1.30) |
| >13 | 1.00 (reference) | 1.00 (reference) |

Notes: PR = prevalence ratio; CI = confidence interval.

^*^ Adjusted for sex, age, marital status, body mass index, smoking habit, alcohol intake, and frequency of going outdoors.

# Table S3. The contribution of number of remaining teeth in explaining the association between socioeconomic status and oral frailty assessed using complete case analysis (n = 15,372)

| **Explanatory variables^a^** | **Mediator** | **TE, PR (95%CI^b^)** | **CDE, PR (95%CI^b^)** | **PE^c^ (%)** |
| --- | --- | --- | --- | --- |
| Equivalized income, $ (ref, >20,000) | Number of remaining teeth: >20 | 1.26 (1.19–1.34) | 1.23 (1.12–1.36) | 11.5 |
| Educational attainment, years (ref, >10) |  | 1.27 (1.19–1.36) | 1.13 (1.00–1.28) | 52.6 |

Notes: 95% CI, 95% confidence interval; CDE, controlled direct effect; PE, proportion eliminated; PR, prevalence ratio; TE, total effect.

All models were adjusted for sex, age, marital status, body mass index, smoking habits, alcohol intake, and frequency of going out.

^a^ Each explanatory variable was separately included.

^b^ Estimated by bootstrap with 1000 replications.

^c^ Proportion eliminated = (PR^TE^ － PR^CDE^)/(PR^TE^ － 1)

# Table S4. The contribution of number of remaining teeth in explaining the association between socioeconomic status and oral frailty based on a different cutoff for the number of remaining teeth

| **Explanatory variables^a^** | **Mediator** | **TE, PR (95%CI^b^)** | **CDE, PR (95%CI^b^)** | **PE^c^ (%)** |
| --- | --- | --- | --- | --- |
| Equivalized income, $ (ref, >20,000) | Number of remaining teeth: >10 | 1.27 (1.22–1.32) | 1.25 (1.18–1.31) | 6.9 |
| Educational attainment, years (ref, >10) |  | 1.30 (1.24–1.35) | 1.26 (1.18–1.34) | 13.0 |

Notes: 95% CI, 95% confidence interval; CDE, controlled direct effect; PE, proportion eliminated; PR, prevalence ratio; TE, total effect.

All models were adjusted for sex, age, marital status, body mass index, smoking habits, alcohol intake, and frequency of going out.

^a^ Each explanatory variable was separately included.

^b^ Estimated by bootstrap with 1000 replications.

^c^ Proportion eliminated = (PR^TE^ － PR^CDE^)/(PR^TE^ － 1)

# Table S5. Robustness to unmeasured confounding (Mediational E-values) of Mediating effect due to number of remaining teeth in the association between socioeconomic status and oral frailty

| **Explanatory variables^a^** | **Mediator** | **Controlled direct effect^b^** | |
| --- | --- | --- | --- |
|  |  | **Mediational E-value for point estimate^c^** | **Mediational E-value for confidence limit^d^** |
| Equivalized income, $  (ref, >20,000) | Number of remaining teeth: >20 | 1.76 | 1.54 |
| Educational attainment, years (ref, >10) |  | 1.74 | 1.43 |

Notes: ^a^ Each explanatory variable was separately included.

^b^ Adjusted for sex, age, marital status, body mass index, smoking habits, alcohol intake, and frequency of going out.

^c^ Mediational E-values of controlled direct effect present the minimum strength of association on the prevalence ratio scale that an unmeasured confounder would need to have with both mediator and outcome to fully explain away the observed controlled direct effect of explanatory variables on outcomes conditional on included covariates.

^d^ Mediational E-values of controlled direct effect for the 95% confident interval limit closest to the null denotes the minimum strength of association on the prevalence ratio scale that an unmeasured confounder would need to have with both mediator and the outcome to shift the 95% confident interval to include the null value conditional on included covariates.
